# Supplementary material for: Management of residents in difficulty in a Swiss general internal medicine outpatient clinic: Change is necessary!
Source: PLoS One. 2021 Jul 20;16(7):e0254336. doi: 10.1371/journal.pone.0254336 (PMC8291751; doi:10.1371/journal.pone.0254336)
Supplement: Attachment — Submitted filename: response to reviewers def.docx [file pone.0254336.s001.docx]

Dear Dr. Ritesh G. Menezes,

Academic Editor PLOS ONE

Thank you for giving us the opportunity to submit a revised version of our manuscript “*Management of residents in difficulty in a Swiss general internal medicine outpatient clinic: changes are needed!*” (ref.no. PONE-D-20-15571) to “PLOS ONE”. We appreciate the time and effort that you and the Reviewers have dedicated to providing your valuable feedback on our manuscript, and we are grateful for your comments on our paper. All suggestions have been incorporated. Here is an itemized, point-by point response to each comment and concern.

| *The manuscript reports qualitative research conducted in 2016. Are the results still relevant in 2020? Revise the manuscript accordingly. The Swiss system would have adopted new measures for the management of residents in difficulty in the intervening time. Discuss the findings in the setting of 2020: explaining what changes have been performed in the years since the interviews were carried out, and how their results can be generalized and used in the present setting.* | Indeed, Thank you for giving us the opportunity to highlight the follow up of this action research process. Since 2015, various processes and training courses that have been developed and implemented at the Faculty of Medicine in Geneva. (lines 489-508) |
| --- | --- |
| *1. Please ensure that your manuscript meets PLOS ONE's style requirements, including those for file naming. The PLOS ONE style templates can be found at*  *https://journals.plos.org/plosone/s/file?*  *id=wjVg/PLOSOne_formatting_sample_main_body.pdf and*  *https://journals.plos.org/plosone/s/file?*  *id=ba62/PLOSOne_formatting_sample_title_authors_affiliations.pdf* | We have revised the PLOS ONE’s style requirements and modified our manuscript in order to meet the criteria. |
| *2. When reporting the results of qualitative research, we suggest consulting the COREQ guidelines: http://intqhc.oxfordjournals.org/content/19/6/349. In this case, please consider including more information on the number of interviewers, their training and characteristics; how participants were selected; if a pilot study was tested; how data was coded; if bias issues were considered. Moreover, please consider providing the interview guide as a supplementary material.* | Thank you for your comment: we have added more information, and provided the interview guide as a supplementary material*.*  Lines 135-161 |
| *3. We suggest you thoroughly copyedit your manuscript for language usage, spelling, and grammar. If you do not know anyone who can help you do this, you may wish to consider employing a professional scientific editing service.* | Thank you for your comment, we have made sure that our manuscript was read and reworked by an English mother tongue researcher.  See Acknowledgments lines 520-522 |
| ***Reviewer #1:*** |  |
| *1. Clarify what is meant by senior resident and how they are different from residents. Also please elaborate on their responsibilities regarding the supervision of residents.* | Thank you for your thorough feedback. As you will see, we have precised their role, and event changed their name in English, after having reviewed how they are specifically named in a few articles. See lines 126-128  We have also explained why they are so important to consider while speaking about supervision Lines 137-141 |
| *2. Elaborate on how the current system identifies residents in difficulty, as it is mentioned that heads of divisions rarely establish the insufficient skills of residents.* | Since recently, it is a faculty wide process that allows learners with difficulties to be identified more quickly, through various methods and tools of identification (supervisors, portfolio, clerkship assessment, etc).  Lines 489-508 |
| *3. In many of the categories, the supervisors’ views and opinions were well-presented; however, the resident’s views were limited. Particularly what residents thought of their supervisors’ abilities and skills in managing residents in difficulty* | Thank you, we agree with your comment and we have therefore more explained why the resident’s view are limited.  The residents reported a taboo when it came to expressing that they were in difficulty. They also tend to trivialize their difficulties, saying that all residents encountered some level of difficulties. Lines 252-258 |
| *4. The units for the information in Table 1 are not clear (e.g. supervision experience and clinical experience). Clarify what is meant by postgraduate title.* | We have rethought the presentation of Table 1 and decided to simplify it to make it clearer. |
| *5. The overall structure and organization of the manuscript should be revised to make reading easier. For example, the part of ethical consideration should be moved.* | We have edited and changed the manuscript to increase the flow.  The ethical consideration have been moved. Line 510 |
| *6. There are issues with formatting that need revision. The legends for figures are found within the body of the manuscript (not the figures themselves). The information written in Box1 can be included in the body of the manuscript and not as a box.* | We paid attention to the figures and their legends.  Following the recommendations of the Editor, we provided the interview guide as a supplementary material. |
| *7. The manuscript would benefit from editing the grammar and punctuation* | Thank you for your comment, we have made sure that our manuscript is read and reworked by an English mother tongue researcher. |
| ***Reviewer #2:*** |  |
| *The purpose of Participatory action research is to enable action. According to Baum et al.*  *"Action is achieved through a reflective cycle, whereby participants collect and analyse data, then determine what action should follow. The resultant action is then further researched and an iterative reflective cycle perpetuates data collection, reflection, and action as in a corkscrew action...The researched cease to be objects and become partners in the whole research process: including selecting the research topic, data collection, and analysis and deciding what action should happen as a result of the research findings." (Journal of Epidemiology & Community Health 2006;60:854-857*  *q*  *In the actual study the iterative reflection cycle and the involvement of the researched objectives (supervisors of residents in difficulty) is not described. --> could you comment on it?* | Thank you for your thorough feedback. As you can see, we have added to our manuscript a few lines in the discussion with a view to clarifying what happened next, once we presented our results in the whole division, but also at the Faculty management level. The abstract has also been modified to be more specific. Lines 489-508 |
| *one of the major limitation of the study is the limited number of focus groups (FG) and the single center context of the stydy that may limit generalization of results --> add a paragraph about limitations* | Thank you, we agree with your comment and we have therefore added a paragraph about strengths and limitations. Lines 466-486 |
| *It may not be clear for the reader why the Chief physician was not involved in FG with attending physicians. What was the aim and the added value to interview separately the Chief physician. Do questions to him differ from other groups?* | Thank you, we agree with your comment and we have therefore described our aim in more details. *Line 149-151* |
| *FG was runned with 6 or 7 participants, which is good. It could be interesting to know how many physicians from the primary care division were eligible to participate to the FG and how the recruitment was performed (do they have volunteered to participate or or a subset of them was recruited?) How many refused to participate or were not invited?*  *CAVE: about the number of participants to FG the text say 6 residents but the table 1 state 7 residents--> please correct* | We have precised our recruitment.  Lines 135-161  Corrected line 185 |
| *FG last about 80 minutes and in these 80 min 7 participants were exposed to 9 questions: hence each participant had a mean of 1.5 minutes to answer, discuss and deepen, counter opinions for other participants for each point, which seem ambitious to an in depth*  *discussion --> could you comment on this ?* | We agree with your concern: to explain this, we can say that on the one hand the perceptions were relatively homogeneous in the groups, and on the other hand that not everyone expressed in detail for each question. In some cases, the participants agreed with the contents already discussed. |
| *At the end of the first paragraph "Summary of results" you say that your results are in line with specialized literature, without specifing what parts of your results are similar and to which articles you refer. Do you mean other aticles using the Fishbein's framework or also studies that used other framework to investigate the problem? It would be interesting to put results more in perspective with the existing literature and the results of other studies. Are there elements that are different from what other groups found?* | We have mentioned research on similar topics that have also used this model successfully.  Lines 392-93 |
| *The local context and the small number of FG raise the question about the generalizability of results outside western Switzerland. How do your results about Attitude and behavioural beliefs, normsand normative belief and self-efficacy and efficacy belief relate*  *to other studies. They are probably related to the local context and culture --> please comment on it* | We agree with your comment, and added a few comments in the Strengths and limitations section.  Lines 468-81 |
| *The study has some limitations (e.g. number of participants, number of FG, monocentric design) that limit the generalisability of results. I suggest Limitations to be adressed in a specific paragraph.* | Thank you, we agree with your comment and we have therefore added a paragraph about strengths and limitations Lines 468-81 |
| *In result section correct the different number of participants in residents FG between text and table 1* | Done. |
| *End of the first paragraph of Discussion: change "but our in depth analyze permits..." to "but our analysis allows..."* | We changed the words. Lines 389-91 |
