# Supplementary material for: Management of residents in difficulty in a Swiss general internal medicine outpatient clinic: Change is necessary!
Source: PLoS One. 2021 Jul 20;16(7):e0254336. doi: 10.1371/journal.pone.0254336 (PMC8291751; doi:10.1371/journal.pone.0254336)
Supplement: Attachment — Submitted filename: response reviewers submission 3 PLOS ONE.docx [file pone.0254336.s002.docx]

**Manuscript ID number**: PONE-D-20-15571R1

**Title of paper**: Management of residents in difficulty in a Swiss general internal medicine outpatient clinic: change is necessary!

| **Reviewer comments** | **Answers** | **Text modified** |
| --- | --- | --- |
| ***Reviewer 1*** |  |  |
| Minor concerns:  Thank you for your response. I enjoyed reading the revised manuscript and I see you addressed the majority of the issues that were raised previously.  There are still minor issues with punctuation and some word choices (e.g. beneficiate line 16) that would benefit from revision. | Thank you for your feedback, we are delighted that the revised manuscript addressed most of the issues you raised.  We have decided to ask an English native to proofread our manuscript and made quite a few changes. We thank you for the suggestion and believe that the changes will clear this minor issue and that is has strengthened the clarity and fluidity of our manuscript. | Please see manuscript for a detailed look at the changes made. |
| ***Reviewer 2*** |  |  |
| *Authors have answered in a satisfactory way to all comments. The articulation of sentences and concepts is more fluid. I have no additional comments* | Thank you for your feedback, it is nice to hear that you are satisfied with our responses. | Please see manuscript for a detailed look at the changes made. |
| **Additional Academic Editor Comments:** |  |  |
| Some errors of English grammar and use still persist in the revised manuscript. | Thank you for your general comment, as mentioned before we have addressed these concerns and ask an English native speaker to proofread our manuscript.  Thank you for the opportunity to publish in your journal and we hope that these last changes strengthened our manuscript and that you will be as happy with its content as we are now. | Please see manuscript for a detailed look at the changes made. |
